# Supplementary material for: Prevalence and burden of bronchiectasis in a lung cancer screening program
Source: PLoS One. 2020 Apr 13;15(4):e0231204. doi: 10.1371/journal.pone.0231204 (PMC7153864; doi:10.1371/journal.pone.0231204)
Supplement: S1 Table — (DOCX) [file pone.0231204.s002.docx]

S1 Table. Post-hoc study for patients older than 55 years and smokers of more than 30 pack-years

Baseline LDCT

|  | Bronchiectasis  N= 163 | Controls  N= 155 | P value |
| --- | --- | --- | --- |
| Subjects without nodules, n (%) | 73 (44.8) | 123 (79.4) | <0.001 |
| Subjects with nodules that do not meet criteria for further test, n (%) | 48 (29.4) | 6 (3.9) | <0.001 |
| Subjects with new nodules, n (%) | 90 (55.21) | 32 (20.6) | <0.001 |
| Subjects with nodules requiring additional tests (false positives), n (%) | 42 (35.5%) | 26 (17.45) | 0.0051 |
| Additional LDCT | 42 (25.77) | 27 (17.42) | 0,071 |
| Indication for antibiotics | 13 (7.98) | 3 (1.94%) | 0.014 |
| Indication for biopsy | 2 (1.23) | 1 (0.65) | 0.592 |
| PET | 3 (1.84) | 1 (0.65) | 0.339 |
| Lung cancer (true positives), n (%) | 4 (2.45) | 1 (0.65) | 0.195 |

Annual LDCT

|  | LDCT with Bronchiectasis  N= 371 | LDCT Controls  N= 311 | P value |
| --- | --- | --- | --- |
| New nodules, n (%) | 59 (15.9) | 34 (10.94) | 0.060 |
| New nodules but do not require  additional tests, n (%) | 11 (2.96) | 4 (1.29) | 0.137 |
| Growth nodules, n (%) | 12 (3.23) | 14 (4.5) | 0.389 |
| Growth nodules but do not require additional tests, n (%) | 2 (16.67) | 4 (28.57) | 0.473 |
| False positives, n (%) | 69 (18.6) | 51 (16.4) | 0.452 |
| Additional LDCT | 64 (17.25) | 42 (13.5) | 0.179 |
| Indication for antibiotics | 29 (7.82) | 17 (5.47) | 0.223 |
| Indication for biopsy | 7 (1.89) | 6 (1.93) | 0.968 |
| PET | 4 (1.08) | 7 (2.25) | 0.226 |
| Lung cancer (true positives), n (%) | 0 | 4 (1.29) | 0.028 |
