# Supplementary material for: Prevalence and burden of bronchiectasis in a lung cancer screening program
Source: PLoS One. 2020 Apr 13;15(4):e0231204. doi: 10.1371/journal.pone.0231204 (PMC7153864; doi:10.1371/journal.pone.0231204)
Supplement: Attachment — Submitted filename: 1. Response to Reviewers.docx [file pone.0231204.s003.docx]

Response to Reviewers

EDITOR:

1. The manuscript meets the style requirements of PLOS ONE, including file name requirements.
2. Funding information does not appear in the Acknowledgments section or other areas of the manuscript.
   *** Funding sources have been withdrawn from the manuscript and specified in the cover letter.** **I would like to change the financing status. The correct Funding Statement is: “The authors received specific funding for this work”**.
3. Please include captions for your Supporting Information files at the end of your manuscript, and update any in-text citations to match accordingly.

*** A table has been added as Suppoting Information.**

Reviewer #1

- Although there is reference to the screening protocol, it would be helpful to outline what is considered "positive."

*** The definition for positive results is explained in the lines 122 and 130. Changes have been made as suggested by the 2nd reviewer so that the information is now more clear.**

- Current standard of care by 2D measurements, which is done in the US (Europe is using more volumetrics), includes a "positive" scan as > or = 6 mm. It appears the analysis was done with 5mm, which seems like a small difference but will alter the numbers, particularly when discussing "false positive." This paper will be referenced for "false positive" but will not be accurate for the current era if using an outdated definition of "positive." It would improve the analysis tremendously to use the updated definition of "positive."

***It is true that the screening studies have used different measurements when considering a positive nodule:**

. NLST (US): > or = 4 mm

. USPSTF (following National Comprehensive Cancer Network, US): > or = 6 mm

. LSS (from NLST): > or = 4 mm

. DANTE (Italy): > or = 6 mm

. DLCST (Denmark): > or = 5 mm

. MILD (Italy): > or = 5 mm

. NELSON (Netherlands / Belgium): volumetric measurements

. ITALUNG (Italy): > or = 5 mm

**In fact, in our I-ELCAP protocol we are already using 6 mm as a cut-off point since 2014. However, this study was previously carried out (follow-up until 2014), with the cut-off point at 5mm also used in other programs. Unfortunately, and although it would be very useful, we do not have the results only in those larger than 6mm, and the indication for additional tests was done according to the current protocol at that time. The aim of this study was to compare the prevalence of nodules in patients without bronchiectasis vs. those without bronchiectasis, according to the current evaluation of nodules at the time of study.**

- Care must be taken with analysis of impact of bronchiectasis within a screening program as the number of cancers is low. As part of this topic, the screening program includes those with a lower risk than those who qualify for screening in the US (starting age 40 with just 10 pack years. This can significantly impact the screening outcomes that does not make the generalizable to the currently qualifying population. If it is possible to separate out the population of those > or = 55 yrs old and pack years > or = 30 to determine if the numbers significantly change. (at a minimum it is important to acknowledge this difference from the currently approved lung screening population as publications like this get referenced to represent expectations within a lung screening program). This is important as "false positive" is something generally being overstated within lung screening programs and is getting broadly confused. This makes it even more important to be very clear about any false positive analysis.

*** We have carried out a posthoc and subgroup analysis. The results have been added on line 324 as well as a table in Supporting Information, and seem to be very similar to the whole group for baseline study. Differences are not as significant on follow-up CTs, although the size of the group is limiting the analysis.**

- Line 308, "the number of subjects with new nodules during follow-up was 15% and 7% for individuals with and without bronchiectasis": What is considered "new nodules"? Does this mean a positive nodule of at least 5 mm? Or does this mean any nodule?
  ***It refers to the percentage of TACS with the presence  of any nodule of any size that does not meet the criteria for suspected malignancy of the I-ELCAP protocol:**

**“Newly identified solid or part-solid nodule < 3.0 mm or nonsolid nodule of any size”**

**It has been clarified in the manuscript on lines 123 and 132.**

**The number refers to CTs and not subjects and has been now corrected in the revised manuscript.**

- The overall analysis of bronchiectasis and lung screening analysis could be more clear.
  ***Some changes have been made with the suggestions of reviewer 2 to make it more clear.** **For example, the results of the different groups have been exposed in an exclusive way to make it more evident.**
- I do not see all the data from the analysis

***The database is currently available in Figshare:** [**https://doi.org/10.6084/m9.figshare.11788836.v1**](https://doi.org/10.6084/m9.figshare.11788836.v1)

Reviewer #2

- Do you have an estimate for Power to detect difference between these groups (i.e. cancer incidence)?

***A prevalence of bronchiectasis of 16% has been described in other lung cancer screening programs. To estimate this prevalence with an accuracy of +/- 1%, an n = 1898 is required.**

**The sample necessary to estimate a difference in the occurrence of false positives, and therefore, the need for additional CTs or other studies, is calculated. The presence of false positives in lung cancer screening studies is 16% in the baseline study and 6% in the follow-up studies. To estimate a difference in the prevalence of the baseline study of 16% +/- 9% of false positives, a sample size of 334 patients is required on each side (power 0.80, alpha 0.05). For the incidence of false positives in follow-up CT scans of 6% +/- 7%, 303 patients are required on each side (power 0.80; alpha 0.05).**

**According to literature recommendations, 95%CI was given to inform better about the differences in proportions for lung cancer.**

**The difference in proportion for lung cancer in baseline study was 0.84% (95%CI: -0.061% to 0.23%) p= 0.256**

**The difference in proportion for lung cancer in annual CTs was -1.84% (95%CI -0.4% to 0.32%) p= 0.094.**

***This data has been added on line 252**

**The sample size calculations before the study are:**

**The sample size to detect a 5-fold risk difference in cancer between the groups is 333 for each group, taking a baseline prevalence of 1%, power 80% and p=0.05.**

**The sample size to detect a 6-fold risk difference in cancer between the groups is 176 for each group, taking a baseline prevalence of 1.4%, power 80% and p=0.05.**

**So, the sample size is not enough to assess risk differences below 5-fold and 6-fold respectively.**

- Line 138 do you mean to say that controls were matched on these variables?

***Yes, thanks for the correction. It has been introduced in this way in the manuscript being clearer.**

- Table 3A: recommend using parallel phrases as described in text. E.g. nodules that do not meet criteria for further tests; nodules requiring additional tests (false positives); lung cancer (true positives).

***It is changed in the manuscript as suggested.  The data has been corrected so that the results are exclusive and better understood.**

- Table 4. Regression analysis to assess appearance of false positives on baseline and follow-up studies. Suggest rewording title to be more descriptive “relationship of variables with false positive nodules”

***Thank you so much for the suggestion. It is changed in the manuscript as suggested.**

- Might be helpful to describe what if any guidelines used for follow up, antibiotics or indicate that testing was done, antibiotics were prescribed, if at the discretion of treating provider or local study team (example, as in line 226 “an indication for antibiotics”).

*** It is added in the manuscript that the tests or treatments have been indicated at the discretion of the attending physician, following the protocol recommendations, line 116. Thank you so much for the suggestion.**

- Table 3 comes after Table 4 and has unclear text and formatting:
  Why is there a “B” without an “A” label? Perhaps a formatting error?

***There is indeed an error and it is unclear. Therefore the tables now follow one another with numbers up to 5, without breakdown by A and B, except for Table 4.**

- Unclear what this means “Growth nodules but do not require additional tests” a growing nodule that didn’t undergo additional testing? That would not be typical

***The protocol specifies the growth criteria that it considers are associated with the indication of additional tests:**

**“Percentage change consistent with malignancy = (diameter at time 2 - diameter at time 1)/ diameter at time 1**

**a) if nodule < 6 mm in diameter, % change > 50% growth**

**b) nodules 6-9 mm, % change > 30% growth**

**c) nodules > 10 mm, % change > 20% growth”**

**It has been clarified in the manuscript on lines 123 and 132.**

- Line 303 – most reports of annual compliance in the US at least, are lower than what the authors report and even lower than what was seen in this study

***Indeed there are other studies with lower adherence and this data has been included in the manuscript, line 304. Thanks for the suggestion.**

- Line 317 – not sure what conclusion you are drawing here (patients with bronchiectasis should not be screened?)

*** Perhaps patients with bronchiectasis that have been seen to need more follow-up and also have worse lung function, should be followed by a pulmonologist in clinical consultations. More studies would be needed to assess whether these patients should follow a different protocol in lung cancer screening given the findings.**

- Line 325 the summary here seems to differ from what is shown in Table 3B. Is table 3A missing (showing prevalence)

***The number have been revised and corrected as indicated.**

- Line 363 suggest adding qualifier for bronchiectasis to improve clarity, for example: “the degree of this limitation is also correlated to with the radiographic severity of bronchiectasis” to be more clear.

***We appreciate the suggestion. It is changed in the revised manuscript.**
